# Fixateure externe zur Behandlung der intrapartalen Symphysensprengung: Fallbericht und Diskussion

**DOI:** 10.1007/s00113-020-00936-x

**Published:** 2020-12-17

**Authors:** M. Müller, F. Greve, M. Zyskowski, M. Wurm, P. Biberthaler, C. Kirchhoff

**Affiliations:** Klinik und Poliklinik für Unfallchirurgie, Klinikum rechts der Isar, Technische Universität München, Ismaninger Straße 22, 81675 München, Deutschland

**Keywords:** Symphysenruptur, Maternales Geburtstrauma, Peripartal, Postpartal, Fixateur externe, Peripartum, Pubic symphysis separation, Peripartum symphysis rupture, Diastasis, External fixation

## Abstract

Die komplette intrapartale Symphysenruptur ist eine seltene, aber schwerwiegende Komplikation der natürlichen Geburt mit einer Inzidenz von 0,03–3 ‰. Kleine Partialrupturen mit geringen Dehiszenzen sind eine Domäne der konservativen Therapie mittels Beckenorthese. Bei größeren symphysären Dehiszenzen sollten eine operative Reposition und Fixierung erfolgen. Im Folgenden wird der Fall einer jungen zweitgebährenden Mutter mit kompletter Symphysensprengung und Dehiszenz von 39 mm beschrieben. Die operative Therapie mittels Anlage eines supraacetabulären Fixateur externe über einen Zeitraum von 12 Wochen lieferte ein gutes Ergebnis.

## Abstract (en)

A complete peripartum pubic symphysis separation is a rare but severe complication of natural birth. Its incidence is estimated to be 0.03–3 ‰. Minor partial separations with a small width can be treated with a pelvic binder. Separations with major dehiscence should be treated by surgical reduction and fixation. This article presents the case of a 30-year-old woman who suffered a complete rupture of the pubic symphysis during the birth of her second child. Radiographic dehiscence was 39 mm. The operative treatment was carried out using a supra-acetabular external fixator for 12 weeks with a good result.

Ligamentäre Verletzungen des Beckens treten am häufigsten nach Hochrasanztraumata wie Verkehrsunfällen und klassischerweise bei verunfallten Motorradfahrern auf. Mütterliche Geburtstraumata des vorderen Beckenrings im Sinne einer vollständig dislozierten Symphyse werden dem Unfallchirurgen nur selten vorgestellt. Gründe sind die seltene Inzidenz sowie die weitverbreitete konservative Therapie insbesondere bei geringfügigen Dislokationen. 

Im vorliegenden Fall wurde eine Therapie mittels Fixateure externe angewandt, wie sie bei hochenergetischen Verletzungen „state of the art“ ist.

## Falldarstellung

### Anamnese

Eine 30-jährige Patientin stellte sich 2 Tage nach der Geburt ihres Kinders in unserer Klinik mit Beschwerden im Bereich des Beckens vor. Unmittelbar nach der Geburt habe sie Schmerzen sowie eine subjektive Instabilität im Bereich des vorderen Beckenringes verspürt. Gehen und Stehen waren nicht mehr möglich. Im Liegen bestand eine Unfähigkeit zur Elevation des Beines von der Unterlage.

Die Geburt in der 40 + 2 Schwangerschaftswoche (SSW) aus vorderer Hinterhauptslage wurde durch die Patientin und die behandelnden Gynäkologen als regelrecht beschrieben. Die Patientin berichtet über forcierte Lagerungstechniken mit passiver Flexion und Abduktion beider Beine im Hüftgelenk, wodurch die Austreibung erleichtert werden sollte. Die beschriebene Technik entspricht dem McRoberts-Manöver, welches ursprünglich für die verbesserte Kindesentwicklung bei Schulterdystokie beschrieben wurde [4]. Es handelte sich um das zweite Kind der Frau. Das Geburtsgewicht lag bei 3820 g, bei einem Kopfumfang von 36,0 cm und einer Länge von 52,0 cm. Das Kind zeigte im Verlauf eine regelrechte Entwicklung. Ein Geburtstrauma seitens des Kindes lag nicht vor.

Bei initialem Verdacht auf eine neurologische Pathologie aufgrund einer klinischen Hüftbeugerschwäche war die Patientin primär den Kollegen der Neurochirurgie vorgestellt worden. Nach Abschluss der bildgebenden Maßnahmen und Diagnosestellung erfolgte die konsiliarische Vorstellung der Patientin in unserer Notaufnahme.

### Befund

In unserer klinischen Erstuntersuchung war die Diagnose der Symphysensprengung aus der MRT-Bildgebung bereits bekannt. Auf die gängige Stabilitätsprüfung des Beckenringes mit Kompression der beiden Beckenschaufeln über den Spinae iliacae anteriores superiores wurde daher verzichtet. Es zeigte sich ein regelrechter inspektorischer Befund ohne wesentliche Schwellung oder Hämatome. Die Patientin war nur transfermobil, Aufstehen und Gehen waren nicht möglich. Peripher-neurologisch zeigte sich ein unauffälliger Befund. Sensibilität wurde allseits regelrecht angegeben. Auch motorisch lagen keine Paresen vor. Eine schmerzbedingte Schwäche der Hüftbeugung wurde initial (vor der bildgebenden Diagnostik) als Parese fehlgedeutet.

Aus gynäkologischer Sicht bestanden ebenfalls keine Auffälligkeiten. Es gab keinen Hinweis auf eine vaginale Verletzung oder Verletzung des Urogenitaltraktes. Blasen- oder Mastdarmstörungen bestanden ebenfalls nicht.

### Bildgebende Diagnostik

**Figure 1:**
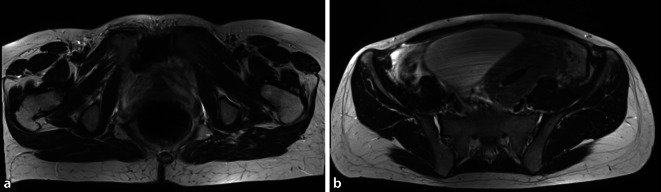

Aufgrund der initialen Verdachtsdiagnose einer neurochirurgischen Problematik am Becken wurde durch die vorbehandelnden Kollegen eine MRT-Bildgebung der LWS und des Beckens veranlasst (Abb. 1). Es zeigte sich eindrücklich ein ventral weit geöffneter vorderer Beckenring mit einer Symphysenweite von 39 mm. Die Iliosakralgelenkfugen stellten sich ventral etwas betont dar. Bei kräftig abgrenzbaren und intakten dorsalen Iliosakralbändern war von einer Instabilität des hinteren Beckenringes MRT-radiographisch nicht auszugehen.

**Figure 2:**
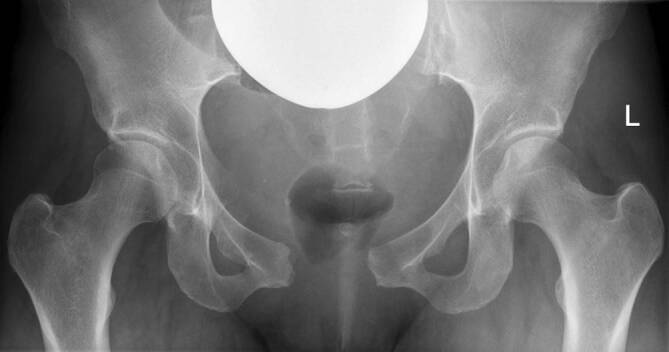

Anschließend erfolgten die konventionelle Röntgenbildgebung (Abb. 2) und die dynamische Untersuchung unter dem Röntgenbildwandler (C-Bogen) in unserer Notaufnahme. Durch manuelle Kompression der beiden Darmbeinschaufeln von lateral ließ sich der vordere Beckenring unter Angabe nur leichter Schmerzen mobilisieren und der Symphysenspalt auf physiologisches Niveau schließen. Um die Reposition zu sichern, wurde ein, in der präklinischen Traumaversorgung üblicher, Beckenkompressionsgurt angelegt.

## Diagnose

Komplette ligamentäre Symphysensprengung mit horizontaler Instabilität des Beckenringes.

## Therapie und Verlauf

Die Patientin wurde zunächst mit angelegtem Beckengurt in die behandelnde Geburtsklinik zurückverlegt, um die kindlichen Erstuntersuchungen durchzuführen und den Wochenbettverlauf zu überwachen. Da unter straffen Beckengurten bereits früh Weichteilkomplikationen (z. B. Ulzerationen und Hautnekrosen) auftreten können, erfolgte die Instruktion zur regelmäßigen Kontrolle des Hautbefundes. Am 4. postpartalen Tag wurde die Patientin zur Verlaufskontrolle vorgestellt. Bei Wiedervorstellung zeigte sich der angelegte Beckengurt trotz ausführlicher Instruktion und kooperativer Patientin disloziert. Die Patientin berichtet über große Probleme bei der Durchführung der hygienischen Maßnahmen bei noch vorhandenem Wochenfluss aufgrund des anliegenden Beckengurtes. In der Röntgenkontrolluntersuchung bei geöffnetem Gurt zeigt sich erneut der weit klaffende Symphysenspalt ohne Tendenz zur Rückbildung.

**Figure 3:**
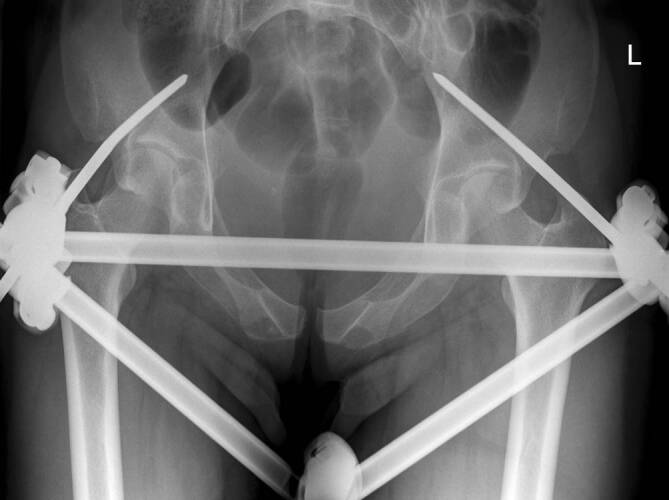

**Figure 4:**
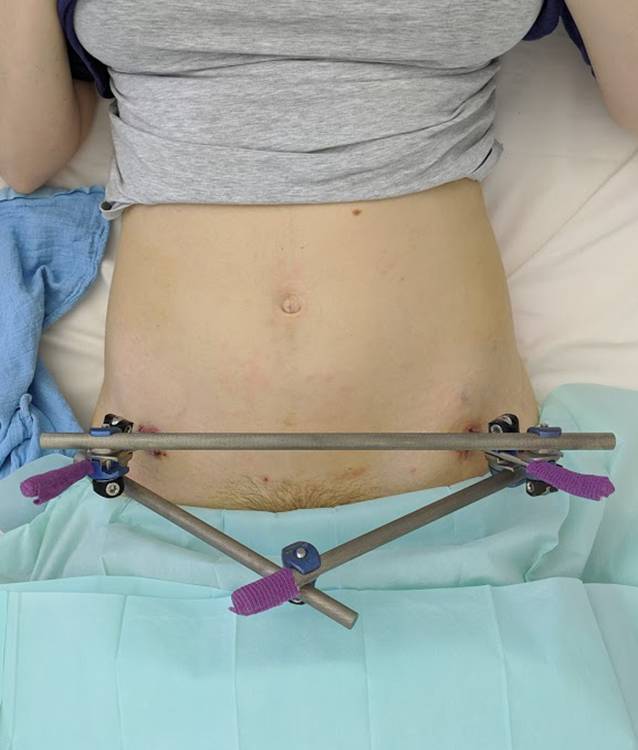

Daher stellten wir die Indikation zur operativen Therapie mittels Fixateure externe. Die Operation konnte am 5. postpartalen Tag komplikationslos in Allgemeinanästhesie durchgeführt werden. Hierzu wurden über minimal-invasive Hautschnitte unter Röntgendurchleuchtung 2 Schanz-Schrauben in typischer supraacetabulärer Position eingebracht. Bei fest verankerten Pins konnte der vordere Beckenring komprimiert werden und der Fixateure externe mittels einfachem Quergestänge vervollständigt werden (Abb. 3). Bei komplikationslosem postoperativem Verlauf wurde die Patientin am 4. postoperativen Tag ins häusliche Umfeld entlassen. Die Belastung wurde auf reine Rollstuhlmobilisation und einfache Transfers mit Abstützen der Beine eingeschränkt. Nach anfänglicher seröser Sekretion aus den Pin-Eintrittsstellen zeigten sich im Verlauf trockene und reizlose Hautwunden (Abb. 4).

Sechs Wochen postoperativ stellte sich die Patientin erneut in unserer Klinik vor, zur Durchführung der ambulanten Metallentfernung des Fixateure externe. In der zunächst durchgeführten präoperativen Röntgenkontrolle zeigte sich ein regelrecht einliegender Fixateur externe ohne Lockerungszeichen oder sekundäre Dislokation. Bei Öffnen der Fixateur-Verstrebung zeigte sich unter Bildwandlerkontrolle jedoch erneut eine Dehiszenz von ca. 2 cm, weshalb auf einen Abbau des Fixateurs zunächst verzichtet wurde. Auch nach 9 Wochen zeigte die dynamische Untersuchung weiterhin eine Restinstabilität.

Das Tragen des Fixateurs und die Ungewissheit über die Stabilität des Beckens stellten für die Patientin eine große psychische Herausforderung dar. Nach ausgiebiger Beratung und Zustimmung der Patientin wurde die Therapie jedoch erneut verlängert.

Letztlich stellte sich die Patientin 12 Wochen postoperativ zur finalen Entfernung des Fixateurs vor. Der intraoperative Befund zeigte nun eine suffiziente Stabilität der Symphyse.

**Figure 5:**
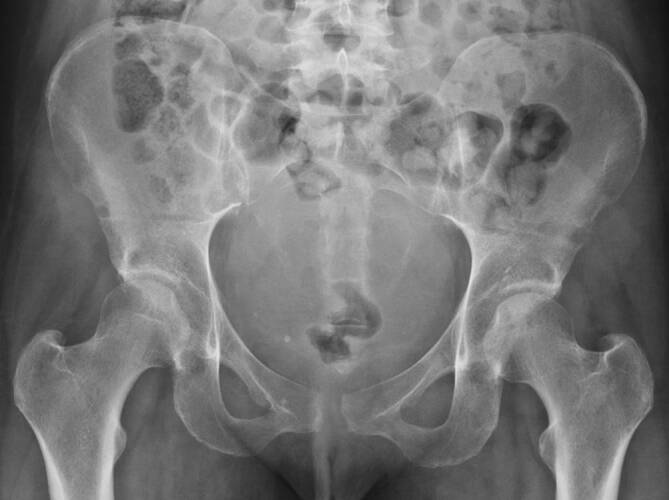

Die anschließende Röntgenkontrolle zeigte eine regelrechte Stellung im Bereich der Symphyse ohne sekundäre Dislokation. Der Symphysenspalt betrug 12 mm (Abb. 5). Der Patientin wurde die zügige Aufbelastung an Unterarmgehstützen erlaubt, sodass sie 10 Tage nach der Metallentfernung ohne Gehhilfen mobil war. Eine stabilisierende Beckenorthese wurde noch für 4 Wochen supportiv empfohlen.

**Figure 6:**
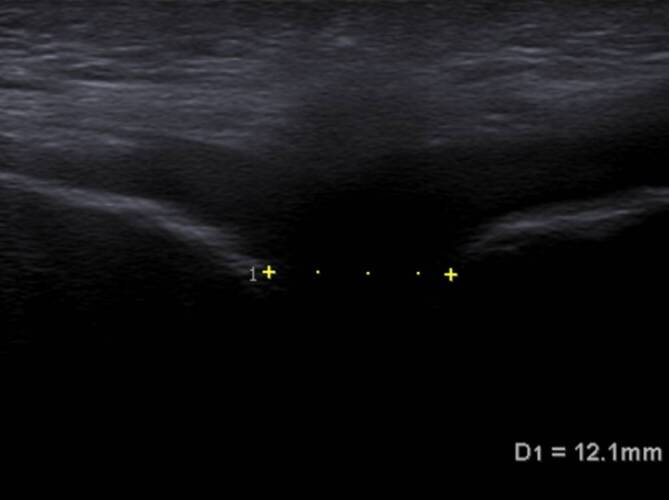

Nach 18 Wochen berichtete die Patientin wieder annähernd beschwerdefrei und uneingeschränkt ihrem Alltag und der Kinderfürsorge nachgehen zu können. In der letzten klinischen Untersuchung 6 Monate nach dem Trauma bestanden ein flüssiges Gangbild, freier Bewegungsumfang in beiden Hüftgelenken und annähernde Schmerzfreiheit. Der Kraftgrad bei der Hüftbeugung betrug 5/5. Die sonographische Kontrolle zeigte eine konstante Weite des Symphysenspalts von 12 mm (Abb. 6) ohne sekundäre Dislokation. Erstmals äußerte die Patientin jedoch sensible Defizite mit Hypästhesie im Bereich des ventralen Oberschenkels und insbesondere in der linken äußeren Labia, welche im Verlauf nur dezent rückläufig seien. Retrospektiv kann nicht eruiert werden, ob diese Beschwerden direkt post partum oder postoperativ nach Anlage des Beckenfixateurs aufgetreten waren.

## Diskussion

Anatomisch stellt die Symphyse das ventralste Bindeglied im Beckenring dar. Die Rr. superiores der Ossa pubica sind Bereich der Symphyse mit einem faserknorpelhaltigen Discus interpubicus verbunden. Stabilisiert wird diese sog. Synchondrose durch das inferiore und das superiore pubische Ligament [17].

Im Rahmen der hormonellen Umstellungen im Rahmen der Schwangerschaft kommt es zu einer physiologischen Weitung des Symphysenspalts [15]. Sonographische Untersuchungen haben gezeigt, dass die mittlere Symphysenspaltweite bei nichtschwangeren Frauen bei 4,07 mm liegt. Im Laufe der Schwangerschaft erhöht sich der mittlere Abstand auf 6,3 mm, wobei in Extremfällen auch Abstände bis 16 mm beschrieben werden. Bei derartigen „symptomatischen Symphysenlockerungen“ treten regelhaft Schmerzsymptome auf. Eine Instabilität des Beckenrings liegt jedoch nie vor. Ein konkreter „Cut-off“-Wert, ab wann eine totale Sprengung der Symphyse mit Bandinstabilität vorliegt, kann allein anhand der gemessenen Weite nicht definiert werden [16].

Im von uns beschriebenen Fall liegt eine extreme Diastase der Symphyse von 39 mm vor. Die klinische Untersuchung sowie die dynamische Untersuchung unter dem Bildwandler bestätigen die Verdachtsdiagnose einer vollständigen Instabilität.

Peripartale Symphysenrupturen sind selten und epidemiologische Daten sehr rar. Entsprechend der Literatur kommt es bei jeder 300. bis 30.000. Geburt zu einer pubischen Diastase [19]. Evidenzbasierte Empfehlungen zur Therapieentscheidung finden sich derzeit nicht. Zum einen besteht die Möglichkeit der konservativen Therapie mit Immobilisation (z. T. strikte Bettruhe) und Anlage eines elastischen Beckengurts für mehrere Wochen. Hierunter konnte in Einzelfällen auch bei ausgeprägten Symphysenspaltweiten (>90 mm) eine spontane Reduktion des Spalts beobachtet werden. Dennoch ist der Genesungsverlauf langwierig, und persistierende Beschwerden werden häufig berichtet [9, 10, 12]. Als operative Therapie stehen die Versorgung mittels Fixateur externe [2] sowie die offene Reposition mit Plattenosteosynthese [5] zur Verfügung.

Aktuell liegen keine hochwertigen prospektiven randomisierten Studien hinsichtlich der Therapieentscheidung vor. In multiplen Erfahrungsberichten wird jedoch eine Symphysenspaltweite von mehr als 25–40 mm als Indikation zur operativen Therapie gesehen [5, 8, 13]. Andere Autoren empfehlen (sofern keine kombinierte Verletzung des hinteren Beckenrings vorliegt) generell die konservative Therapie mit Analgesie, Physiotherapie und Beckengurt. Nur bei persistierenden Beschwerden über 4 bis 6 Monate hinaus wird hier eine operative Therapie mittels Plattenosteosynthese empfohlen [7].

Die Versorgung mittels Plattenosteosynthese wird gemeinhin als Verfahren der Wahl angesehen. Die Anwendung des Fixateur externe dagegen wird vornehmlich als Alternative bei Kontraindikationen gegenüber einen internen Versorgung gesehen [18]. Als Nachteile der Versorgung mittels Fixateure externe werden Pin-Lockerung, Pin-Infekt und Belastung des Patienten genannt.

Aus unserer Sicht kann die Indikation zur primären operativen Versorgung mittels Fixateur externe großzügiger gestellt werden. Der Hauptvorteil liegt v. a. in einer suffizienten Reposition und zuverlässigen Ausheilung mit geringer Symphysenspaltweite ohne die Risiken des operativen Zugangstraumas bei offener Reposition und Plattenosteosynthese. Zudem besteht ein Vorteil in der früheren Mobilisation im Vergleich zu konservativen Verfahren mit Beckengurt. Die mechanische Fixierung des Beckens ohne Beeinträchtigung der Hüftgelenke bietet zudem Vorteile bei der Durchführung von Hygienemaßnahmen im Rahmen der Lochien (Wochenfluss).

Durch den verbreiteten Einsatz von externen Fixateuren in der Primärversorgung von Traumapatienten sowie in der rekonstruktiven Knochenchirurgie stellt ein Fixateure derzeit ein sicheres und komplikationsarmes Verfahren dar. Insbesondere bei Langzeitanwendung kann der Einsatz von Hydroxylapatit(HA)-beschichteten Schanz-Schrauben erwogen werden. Hierdurch kann die Stabilität gesteigert und die Pin-Lockerung-Rate gesenkt werden [14]. Auch Pin-Infektion-Raten lassen sich durch die Verwendung einer HA-Beschichtung reduzieren [11].

Die beschriebenen Sensibilitätsstörungen entsprechen dem Versorgungsgebiet der Rr. cutanei femorales anteriores des N. femoralis am ventralen Oberschenkel bzw. den Gebieten der Rr. labiales anteriores et posteriores aus N. ilioinguinalis bzw. N. pudendus. Aufgrund des anatomischen Nervenverlaufes ist eine intraoperative Nervenschädigung bei Pin-Implantation unwahrscheinlich. Jedoch existieren mehrere Kasuistiken, die Symphysensprengungen und ähnliche begleitende Nervenschädigungen nach Durchführung des McRoberts-Manöver beschreiben. Konkret wurden transiente Ausfälle des N. femoralis sowie des N. cutaneus femoris lateralis beschrieben. Als Pathogenese wird eine Quetschung der femoralen Nerven unter dem Leistenband im Rahmen der forcierten Hüftbeugung und Abduktion vermutet [3, 6].

Die Behandler und die Patientin zeigten sich mit dem Verlauf und dem Ergebnis der operativen Therapie zufrieden. Essenziell ist in jedem Falle eine ausführliche Aufklärung der Patientinnen über das mehrwöchige Tragen des externen Fixateur. Es muss vermittelt werden, dass trotz des ungewohnten Anblicks ein weitgehend normales Leben mit dem Fixateur möglich ist. Durch eine strukturierte Patienteneinweisung in das Leben mit externem Fixateur lassen sich die Patientenzufriedenheit steigern und Komplikationen minimieren [1].

Basierend auf der spärlichen Literaturlage und auf den Erfahrungen in unserer eigenen Praxis empfehlen wir eine Behandlungsdauer mit Fixateur externe von mindestens 10 bis 12 Wochen. Aufgrund der großen Kraftübertragungen und des postpartalen Hormonhaushalts mit physiologischer Lockerung des Bandapparats ist zu einem früheren Zeitpunkt scheinbar keine ausreichende Stabilität gewährleistet.

## Fazit für die Praxis

- Intrapartale Symphysenverletzungen sind selten, und evidenzbasierte Therapiealgorithmen sind noch ausstehend.
- Gering-symptomatische Symphysendiastasen können konservativ mittels Immobilisation und Beckengurt therapiert werden.
- Symptomatische Symphysensprengungen mit relevant vergrößerter Spaltweite profitieren von einer operativen Therapie.
- Der Fixateur externe mit supraacetabulären Pins stellt ein sicheres und minimal-invasives operatives Verfahren dar.
- Die Behandlungsdauer sollte mindestens 10 bis 12 Wochen betragen.
